# Primary Care Providers’ Perspectives on Barriers and Facilitators to Human Papillomavirus (HPV) Vaccination among Young Adults: A Qualitative Study

**DOI:** 10.1007/s13187-025-02802-z

**Published:** 2026-01-07

**Authors:** Coralia Vázquez-Otero, Heather N. Owens, Mariana Arevalo, Joyce Cui, Melody N. Chavez, Melinda L. Maconi, Carley Geiss, Kea Turner, Susan T. Vadaparampil, Veronica Barrios-Monroy, Alissa Pena, Junmin Whiting, Shannon M. Christy

**Affiliations:** 1 Department of Public Health, College for Health, Community and Policy, University of Texas at San Antonio, San Antonio, TX, USA; 2 Department of Health Outcomes and Behavior, Moffitt Cancer Center, Tampa, FL, USA; 3 Morsani College of Medicine, University of South Florida, Tampa, FL, USA; 4 Participant Research, Interventions, and Measurement Core, Moffitt Cancer Center, Tampa, FL, USA; 5 Division of Health Systems, Policy, and Innovations, University of North Carolina at Chapel Hill School of Nursing, Chapel Hill, NC, USA; 6 University of North Carolina Chapel Hill Lineberger Comprehensive Cancer Center, Chapel Hill, NC, USA; 7 Center for Immunization and Infection Research in Cancer, Moffitt Cancer Center, 12902 Magnolia Drive, Tampa, FL, USA; 8 Non-Therapeutic Research Office, Moffitt Cancer Center, Tampa, FL, USA; 9 Department of Biostatistics & Bioinformatics, Moffitt Cancer Center, Tampa, FL, USA; 10 University of California Los Angeles, Los Angeles, CA, USA; 11 University of South Florida, Tampa, FL, USA

**Keywords:** Human papillomavirus, HPV vaccine, Provider recommendation, Consolidated framework for implementation research

## Abstract

Young adults (18–26 years) are at high risk of human papillomavirus (HPV) exposure. Yet, HPV vaccine uptake is suboptimal among young adults. Provider recommendation is frequently the most influential factor in HPV vaccine acceptance. Evidence-based strategies are needed to facilitate provider recommendations for young adults. To inform provider-level strategies for recommending the HPV vaccine to young adult patients, the current study aims to understand current practices and barriers and facilitators to both recommending the HPV vaccine for young adults and for young adults to receive the HPV vaccine. Primary care providers (e.g., physicians, nurses; *n* = 15) in the United States completed a semi-structured interview guided by the Consolidated Framework for Implementation Research (CFIR). Data were analyzed thematically using Nvivo. Most participants identified as female (80%), White (67%), and non-Hispanic (93%). Participants reported varying their HPV vaccine discussions based on visit type (acute vs. wellness) and if the patient is new or established in their practice. Participants reported barriers to HPV vaccination in young adults across several CFIR domains including outer setting (e.g., transient living situation), inner setting (e.g., lack of availability of vaccine in clinic), individual (e.g., sex differences), and process (e.g., burnout). Facilitators included considering the HPV vaccine as a high priority and scheduling future vaccine appointments at the current visit. Evidence-based interventions to increase HPV vaccine uptake among young adults are needed. Healthcare providers identified multilevel barriers to both recommending and delivering the HPV vaccine to their young adult patients.

## Introduction

Although the human papillomavirus (HPV) vaccine is a highly effective cancer prevention tool [1, 2], only 47.4% of United States (US) young adults (18–26 years old) reported receiving one or more HPV vaccine doses [3]. Physician recommendation among this age group has been shown to increase HPV vaccination [4, 5]; however, consistency and quality of provider recommendations varies [6–8]. Few interventions targeting healthcare providers to support their recommendation of the HPV vaccine exist and most focus on those who treat adolescents [9]. However, promoting HPV vaccine uptake among 18 to 26-year-olds is a crucial cancer prevention strategy [10] given their high risk for exposure to HPV and their ability to make their own health care decisions [11, 12]. Using the Consolidated Framework for Implementation Research (CFIR) [13], this study explores current HPV vaccine practices and identifies barriers and facilitators for recommending and delivering the HPV vaccine to young adults.

## Methods

This work is part of larger study aimed at informing HPV vaccine intervention strategies through a survey of young and mid-age US adults [14], interviews with young adults [15], and interviews with healthcare providers (data presented here). We conducted qualitative interviews informed by CFIR (2009), an implementation science framework that considers contextual factors affecting implementation in five domains [13]. Such domains related to implementation of HPV vaccination interventions include: (1) characteristics of individuals (e.g., audience’s understanding of HPV vaccination and its benefits), (2) process (e.g., ways to encourage clinicians to recommend HPV vaccination), (3) inner setting (e.g., infrastructure), and (4) outer setting (e.g., policies that impact vaccination) [13]. CFIR was updated in 2022 [16] to the following domains: (1) Innovation, (2) Outer setting, (3) Inner Setting, (4) Individuals, and (5) Implementation Process. The team considered this update and included it as part of the data analysis process.

### Participants

Healthcare providers who deliver medical care services to young adults ages 18–26 years in US primary care settings were asked about barriers and facilitators to HPV vaccination in young adult patients and implementation of HPV vaccine intervention strategies. Eligibility included: (1) being a US healthcare primary care provider for young adult patients (e.g., primary care, family medicine, and/or OBGYN), (2) aged 21 years or older, (3) English proficiency, (4) access to the internet and/or telephone, and (5) willingness to be audio recorded.

### Recruitment and Data Collection Procedures

Participants were recruited using English-language flyers distributed in-person, via social media (e.g., LinkedIn), via email to health care organizations, clinics, health centers, and community organizations, and via emails and flyers shared with providers identified as working in health care settings across the US through publicly-available email addresses. Attempts were made to recruit individuals from a variety of US states, regions, and settings. Interested individuals contacted the study team. The team screened potential participants via phone, reviewed an information sheet with those who were eligible, and answered questions about the study. The Moffitt Scientific Review Committee and the Institutional Review Board (IRB) of record (Advarra) approved a waiver of signed informed consent.

Following consent, participants were sent an email containing a link to a REDCap questionnaire which assessed sociodemographic [17] and role characteristics (e.g., age, gender, race, ethnicity, clinic role, vaccination role, populations and age groups served, number of patients aged 18–26 years seen in a typical week, and number of years providing care to patients aged 18–26 years) and HPV vaccination discussion practices (i.e., whether patient-provider discussions varied based on patients’ marital status, gender, sexual orientation, type of visit, new versus established patient status, and strategies used to guide discussions with their young adult patients) (adapted from Marzán-Rodríguez et al., unpublished data).

Qualitative interviews were conducted via phone or videoconferencing software by staff with expertise in qualitative methods (MM, CG, and MC) between April and October 2022. The interview guide (adapted from Marzán-Rodríguez et al., unpublished data) was designed to elicit perspectives and practice patterns based upon the original CFIR constructs [13] including *Inner Setting* (e.g., HPV vaccine practices, benefits and challenges of vaccinating young adults in their clinics, and leadership support for HPV vaccination), *Characteristics of Individuals* (e.g., what they believe young adults understand about the HPV and how young adults react when discussing the HPV vaccine), *Process* (e.g., how young adults can be encouraged to be vaccinated and how medical providers can be encouraged to recommend the vaccine to young adults), and *Outer Setting* (e.g., perceived barriers to HPV vaccine initation and completion among young adults). Participants received a $50 gift card.

### Data Analysis

Participant characteristics were summarized using descriptive statistics (Stata version 13). Qualitative data were professionally transcribed verbatim. A codebook with a priori codes were developed from the CFIR informed interview guide questions [18]. Guided by tenants of applied thematic analysis [19], two qualitative researchers (MNC and MM) conducted the initial round of coding of all transcripts independently using NVivo version 12 Plus software. The analysts met regularly to resolve discrepancies and discuss emergent patterns. Findings were then summarized by CFIR domains, and exemplary quotes were selected. As CFIR was updated in 2022 [16], team members (CVO, HNO, SC, KT) reviewed findings and recategorized them based on the updated CFIR. Rigor and trustworthiness were achieved through adherence to standards described by Nowell et al. [20].

## Results

### Participant and HPV Vaccination Discussion Characteristics

Participant characteristics (*n* = 15) are described in Table 1. The average age of participants was 44 (± 12.3) years old. Most were female (80%) and identified as Non-Hispanic White (67%). Only one participant identified as Hispanic. Participants reported that their discussions varied for an acute visit versus a wellness visit (80%), and whether the patient was new or established (67%) (Table 2). Participants reported varying their conversations based upon patient demographics; 40% adjusted their conversations based on gender and 33% based on sexual orientation or gender identity.

Barriers and facilitators to HPV vaccine recommendations and delivery to young adults.

Participants discussed barriers and facilitators to recommending and delivering the HPV vaccine to young adults. Table 3 includes exemplary quotes using both the original CFIR and updated CFIR domains [13, 16].

#### Outer Setting

Participants reported factors external to their clinical practice setting that affected their ability to recommend the HPV vaccine for young adults. The COVID-19 pandemic was discussed as a barrier to HPV vaccination in multiple ways, including being seen a competing priority and contributing to decreased trust in vaccines.

##### Cosmopolitanism

Participants whose practice did not deliver the HPV vaccine reported awareness of locations that did administer the vaccine and reported recommending their patients obtain the vaccine at those locations. Moreover, participants indicated that even if clinics could vaccinate their patients with the HPV vaccine, having alternative sites convenient for the patients could increase completion of the vaccine series amongst young adults. Participants noted that allowing young adults to receive subsequent doses of the vaccine at local pharmacies closer to where they reside could be a facilitator.

##### External Policies and Incentives

Participants indicated that the cost of the HPV vaccine can be prohibitive for uninsured young adults. Moreover, coordinating payment from insurance companies for the HPV vaccine can be complicated in certain settings.

##### Patients’ Needs

Participants described that most young adults visit the clinic when seeking care for an acute condition (rather than for preventive care), and that therefore, vaccines might not be a topic of discussion. Participants also noted that young adults often move (e.g., following school graduations), which might make initiating or completing the HPV vaccine series more difficult (e.g., lack of a consistent provider).

#### Inner Setting

##### Relative Priority

Participants considered the HPV vaccine a high priority in their clinic and reported recommending vaccination to unvaccinated patients during routine visits. Participants described the need to be proactive in training and focusing on teamwork to identify opportunities for HPV vaccination. Scheduling appointments for subsequent doses at the previous appointment to proactively address relying on patient reminders to schedule the follow-up doses was mentioned. Some participants reported that their clinic had standing orders or the capacity to administer the vaccinations at their site.

Among those who did not have the HPV vaccine available for administration at their clinic site, participants stressed that they still recommended the HPV vaccine and wrote the prescription for the vaccine to be received elsewhere (e.g., nearby pharmacy). Few participants reported that their clinic was in the process of expanding their services to provide the vaccines on-site in the future.

##### Organizational Incentives and Rewards

Meeting clinic metrics was noted as a facilitator. Some participants mentioned the possibility of institutional or organizational incentives and rewards for meeting metrics regarding HPV vaccination rates.

#### Characteristics of Individuals

Participants reported HPV vaccine benefits including cancer prevention, fewer positive HPV test results, and a decrease in genital warts diagnoses. Participants also shared their perceptions of sociodemographic differences of patients received the HPV vaccine and those they believed would be more likely be receptive to receive the HPV vaccine.

##### Perceptions of the role of patients’ sex assigned at birth

Participants noted that the initial emphasis on cervical cancer prevention when the HPV vaccine was introduced and limited discussion of other types of HPV-related cancers led to differences during conversations about the HPV vaccine. Participants described that HPV-related conversations are more common during gynecological appointments. Many participants noted that women expressed fear of cervical cancer, while most men sought vaccination to prevent genital warts or protect female sexual partners. Due to the initial approval of the HPV vaccine for females, participants emphasized the importance of clinician recommendations and increased awareness for males. Participants highlighted the value of HPV vaccine campaigns clarifying eligibility for males, noting their effectivenes as most young adult men were vaccinated or receptive to being vaccinated in recent years.

##### Perceptions of the role of patients’ sexual orientation

Four clinicians also mentioned the role of sexual orientation in receptivity of getting the HPV vaccine. One indicated their belief that lesbian, gay, bisexual, transgender, queer, inter-sex, asexual and other identities (LGBTQIA+) patients may be more receptive to receiving the HPV vaccine and that sexual and gender minority patients may be knowledgeable about sexual health due to having received information about HIV. Other clinicians revealed worry that men who have sex with men might not be aware of the benefits of the HPV vaccine.

##### Perceptions of the role of parental influence

Participants discussed how young adults may still be influenced by their parents’ beliefs or prior decisions regarding vaccines and that this may impact HPV vaccination uptake.

#### Process

##### Executing

Participants reported implementation challenges with recommending the HPV vaccine to their patients. Among those, time was noted as a barrier to seeking out HPV vaccine training. Participants also discussed difficulties with determining vaccination history for young adults (not having access to prior records).

## Discussion

Guided by CFIR [13, 16], this study examined barriers and facilitators to HPV vaccination among young adults to foster effective HPV vaccination recommendations and delivery. Findings showed that healthcare providers vary HPV discussions with patients based on the type of visit and if the patient is new or recurring. Discussions also varied based on patient’s gender and sexual orientation or gender identity, as noted in the interviews and the survey data. This is concerning due to the increased rate of HPV-related oropharyngeal cancer in males [21] and the significantly lower HPV vaccine coverage among males [3].

Several barriers to HPV vaccination were identified across CFIR domains. External factors such as the COVID-19 pandemic was identified as a barrier. Previous research using CFIR found that during the COVID-19 pandemic, vaccine hesitancy was also an outer setting barrier to HPV vaccination among adolescents [22]. From 2018 to 2019, there was an increase in HPV vaccination rates among young adults; however, no changes were seen from 2019 to 2022, the period that included the COVID-19 pandemic [3].

Another *outer setting* barrier specific to the young adult population, especially college students, is the transient living situation and lack of routine medical care. Many young adults do not regularly see a healthcare clinician and attend medical appointments when necessary [23]. While some participants noted that receiving care at clinics not offering the vaccine on-site was a barrier, they mentioned that expanding the settings (e.g., local pharmacies) where young adults could initiate the series or receive subsequent doses could increase HPV vaccine uptake. Additional HPV vaccination settings, such as pharmacies [24] and college campuses represent opportunites for HPV vaccination. Indeed, some prior interventions have focused on increasing HPV vaccination in the college setting [25]. For instance, two interventions implemented in US-based colleges included student-facing materials providing education on HPV vaccination and provider-facing training to recommend the vaccine to young adults [26]. Additionally, one intervention included EMR prompts for discussing the HPV vaccine [26, 27]. Both studies reported an increase in HPV vaccination during the study periods [26, 27]. Further research is needed to explore these settings for young adults.

Despite noting a need for more training and teamwork, participants mostly described facilitators under the *inner setting* construct, such as prioritizing the HPV vaccine and scheduling future vaccine dose appointments during initial visits. Scheduling the next vaccination visit at the current visit is recommended to improve the quality of immunization services as part of the Centers for Disease Control and Prevention’s Assessment, Feedback, Incentives, and eXchange (AFIX) program, which aims to improve vaccination rates through quality improvement methods [28].

Regarding the *characteristics of the individuals*, a barrier discussed was how the initial vaccine approval for females has continued to result in lower vaccination trends among males. This is evident throughout the literature with lower HPV vaccine knowledge, awareness, and uptake among males compared to females [29]. Few interventions have been developed to increase vaccination rates among young adult males specifically. An example of an intervention that did so aimed at improving HPV vaccination among young adult males targeted 18–25-year-old gay, bisexual, and other men who have sex with men [30]. The intervention, guided by the Protection Motivation Theory, Information-Motivation-Behavioral Skills Model and the Minority Stress Model, reported higher vaccine initiation among the intervention group compared to the control group [30]. Although not focused on young adults, another intervention used CFIR to guide intervention strategies to increase HPV vaccination in pediatric primary care practices. That study determined the primary barriers to vaccination and developed intervention strategies using the Behavior Change Wheel and Theoretical Domain Framework based on the identified barriers [31]. Future interventions might evaluate similar CFIR-informed approaches to improve HPV vaccination rates among young adults. Facilitators under this construct included the level of knowledge and awareness healthcare providers showed when discussing the benefits of the HPV vaccine, and its value as both a sexually transmitted infection prevention tool and a cancer prevention tool.

Research exploring the effect of parental influence on HPV vaccine uptake among young adult college students found that those who were vaccinated reported their parents were an influencer in their decision while providers had no significant impact on their decision [32]. Since some of the study participants of the current study were employed in colleges/universities, this finding seems to be relevant in that context and time when young adults are transitioning to more independence in their decision-making process [33]. Additionally, among young adults, vaccine dicisions may be influenced by parents if the young adult patient is still on their parents’ insurance and potentially fearful of parents learning about them receiving the vaccine.

Lastly, among the implementation challenges for recommending the HPV vaccine to young adults, providers’ limited availability or time was a barrier HPV vaccine training/education under the *Process* domain. Web-based interventions providing support for behavior modification and skills to recommend the HPV vaccine [34] could address this barrier, and facilitate access and engagement among healthcare providers.

## Strengths and Limitations

The strengths include using CFIR, a framework used to explore implementation barriers and facilitators at multiple levels and differing settings [13], to guide the study and the data analysis. Additionally, mapping the results to the updated CFIR [16] added analytical depth and strengthened the methodology. Furthermore, findings can inform other researchers integrating the original and updated CFIR. Despite these strengths, some limitations should be noted. Most participants were females and identified as non-Hispanic White, which may limit generalizability. As about half of the participants provided care to college/university students, perspectives from healthcare providers serving with non-college young adults should be explored. Future research should determine barriers and facilitators to vaccinating for young adult patients across more diverse clinician populations (e.g., gender, race/ethnicity, settings). Overall, findings from this study can be used to support the development multilevel interventions to increase HPV vaccine uptake among young adults by addressing the barriers identified and building on the facilitators.

## Conclusion

Barriers and facilitators to increase HPV vaccine recomendation and delivery among young adults were identified using CFIR. Previous research using CFIR to analyze barriers and facilitators to implementing HPV vaccine has primarily focused on interventions for adolescent patients. Healthcare providers recommending and delivering HPV vaccines to young adult patients need training and support to confidently discuss the vaccine with their young adult patients. Multilevel interventions to increase HPV vaccine uptake among young adults are needed.

## Figures and Tables

**Table 1:** Participant characteristics (*N* = 15)

Characteristic	*N* (%)
Age	
Mean (SD)	44.2 (12.3)
Range	27–67
Gender	
Female	12 (80.0)
Male	3 (20.0)
Race/Ethnicity	
White	10 (66.7)
Black/African American	3 (20.0)
Asian	1 (6.6)
More than one race	1 (6.7)
Ethnicity	
Non-Hispanic	14 (93.3)
Hispanic	1 (6.7)
Clinic role*	
Pediatrician	2 (13.3)
Family physician	3 (20.0)
Other type of physician	2 (13.3)
Physician Assistant	1 (6.7)
Nurse Practitioner	3 (20.0)
Nurse	4 (26.7)
Vaccination role – schedule appointments	
Yes	3 (20.0)
No	12 (80.0)
Vaccination role – prescribe vaccines	
Yes	14 (93.3)
No	1 (6.7)
Vaccination role – answer patients’ questions about vaccines	
Yes	14 (93.3)
No	1 (6.7)
Vaccination role – administer vaccines	
Yes	8 (53.3)
No	7 (46.7)
Vaccination role – document vaccines	
Yes	7 (46.7)
No	8 (53.3)
Vaccination role – order vaccine supply	
Yes	2 (13.3)
No	13 (86.7)
Vaccination role – other	
Yes: Suggest vaccines used	1 (6.7)
No	14 (93.3)
Years working in this role or similar role	
Less than 1 year	2 (13.3)
1–4 years	3 (20.0)
5–9 years	2 (13.3)
10–14 years	1 (6.7)
15–19 years	3 (20.0)
20 or more	4 (26.7)
Age groups served by practice 0–17	
Yes	7 (46.7)
No	8 (53.3)
Age groups served by practice 18–26	
Yes	15 (100)
No	0
Age groups served by practice 26–45	
Yes	11 (73.3)
No	4 (26.7)
Age groups served by practice 46–65	
Yes	6 (40.0)
No	9 (60.0)
Age groups served by practice ≥66	
Yes	5 (33.3)
No	10 (66.7)
Works directly with the following population:	
All	3 (20.0)
College/university students	8 (53.3)
OBGYN	1 (6.7)
Missing	3 (20.0)
Number of patients ages 18–26 do you see in a typical week	
0	0
1–4	1 (6.7)
5–9	4 (26.7)
10–14	2 (13.3)
15–19	2 (13.3)
20 or more	6 (40.0)
Years providing care to patients ages 18–26	
Less than 1 year	1 (6.7)
1–4 years	3 (20.0)
5–9 years	3 (20.0)
10–14 years	1 (6.7)
15–19 years	2 (13.3)
20 or more	5 (33.3)

* 4 participants indicated more than one response

**Table 2:** Patient-clinician discussion characteristics

Patient-clinician discussions vary based on whether …	*N* (%)
It is an acute vs. well visit	
Yes	12 (80.0)
No	3 (20.0)
Not sure	0
This is a new patient you have only met once vs. an established patient	
Yes	10 (66.7)
No	5 (33.3)
Not sure	0
The patient is single vs. in a committed relationship	
Yes	5 (33.3)
No	10 (66.7)
Not sure	0
The patient is male vs. female	
Yes	6 (40.0)
No	8 (53.3)
Not sure	1 (6.7)
The patient is member of a sexual or gender minority group	
Yes	5 (33.3)
No	10 (66.7)
Not sure	0

**Table 3:** Exemplary quotations from clinicians organized by the original CFIR (2009) and recategorized to the updated CFIR 2.0 (2022)

Exemplary quote	Original CFIR Construct (2009)	Updated CFIR Construct (2022)
**OUTER SETTING** (e.g., the setting in which the primary care organization exists)		
*P: I would say yeah, I would say yeah, because our attention got pulled so hard towards COVID, patients came in with that on their mind. Not just that, but other things as well. But that kind of takes away from some of the preventive things that we would normally do. I would venture to guess that our preventive measures dropped during COVID ‘cause actually we’re trying to work on a project to look into right now. – family physician*	*Outer setting*	*Process*: Assessing context
*P: Well, I think public health should be involved more. Insurance companies have a duty because if they don’t help with the vaccine promotion they’re going to be paying for cancers. So, I think public health and insurance companies can also share some of this burden. – nurse practitioner*	*Outer setting*: Cosmopolitanism	*Outer setting*: Partnerships and connections
*R: Does your clinic provide the HPV vaccine to young adults?* *P: No. The way we do it here is, if we have a student that’s interested in receiving that, I will send them a prescription to the local [pharmacy name], which is right down the stress, less than a mile. They will pick it up, and return back to the clinic, and that nurse would administer*. *P: And they would come back to Student Health Services with the vial and then we’re able to administer. – nurse practitioner*	*Outer Setting*: Cosmopolitanism	*Outer setting*: Partnerships & Connections
*They need an email reminder and a phone reminder and all those things like, “Hey, don’t forget to come in!”… If we could work with pharmacies so that somebody comes in and gets their dose… but they live 45 minutes away, I think it would be more likely for them to come back for dose number two in two months, if they could go get dose number two at [pharmacy name] that’s a few minutes from their house… but we can’t coordinate that. – physician (OBGYN)*	*Outer Setting*: Cosmopolitanism	*Outer setting*: Partnerships & Connections
*So, a lot of times we are still having a bit of insurance coverage, trying to get students vaccinated. We do have some that are free vaccine but then we have others where the students will pay a fee and then they will submit it to their insurance company, so sometimes there is a little bit of a reimbursement issue. – nurse practitioner*	*Outer Setting*: External policies and incentives	*Outer setting*: Financing
*So, again, graduation and moving out of the area. Even though we’ll set up appointments and we’ll send them reminders, these kids get so focused on their academics, they just kind of – they miss appointments all the time and we’re chasing them, chasing them…I just think it’s the age group. They’re just such spontaneous thinkers. – nurse practitioner*	*Outer setting*: Patients’ Needs	*Individuals*: Innovation Recipients
*So, I think also you’re kind of used to the US schedule of vaccines, so you just assume that someone got it, and 18-year-olds that are kind of going from urgent care to urgent care for their visits and they don’t have a primary, they may get missed. So, it’s important for there to be not just your primary but urgent cares, clinics, places like student health. – nurse practitioner*	*Outer setting*: Patients’ Needs	*Individuals*: Innovation Recipients
**INNER SETTING** (e.g., the primary care setting where vaccination is delivered)		
*I don’t have data to tell you for sure, but I know for getting people to get fully vaccinated on this, probably one of the biggest barriers that we have is not doing reminders and already going ahead and scheduling. […]That’s what our clinic does, is they come in, they get their shot, and they leave with the appointment for the next one so it’s already on the books and they have it in their phone. And it’s less work for them to do. So, you could have something like that as opposed to the patient reminders. – physician (OBGYN)*	*Inner setting*: Relative priority	*Inner setting*: Structural characteristics
*Really, I think the biggest incentive is for – to meet the clinical metrics. So, if the clinic itself were incentivized to chase after that metric and make it one of the ones that we’re looking out for that year, I feel like that would kind of give everybody a kick in the pants to do it. – family physician/resident*	*Inner setting*: Organizational Incentives & Rewards	*Inner Setting*: Incentive Systems
*R: To what extent does the leadership at your practice or clinic support HPV vaccination efforts? P: Yeah, so I think they’re very supportive. We’ve ever a teaching specifically about HPV but just vaccination in general, we’re pro-vaccine. – family physician*	*Inner Setting: Culture and Relative priority*	*Inner Setting*: Culture
**CHARACTERISTICS OF INDIVIDUALS** (e.g., roles and characteristics of patients)		
*R: What are some of the benefits to your practice, or to the clinic, if rates of HPV vaccine increases among young adults*. *P: Decreased risk of cancer*. *R: Okay. Anything else?* *P: It might decrease the visits for genital warts … But overall*, *decrease cancer cases. – family physician*	*Characteristics of Individuals*: Knowledge and beliefs about the intervention	*Innnovation*: Evidence base
*I have to say this is my own, indirect studies per se – from when I first started, we’ve seen a lot of young women with genital warts, and now it’s minimal. I talk to my colleagues sometimes to ask her if she’s seen any, and just a drastic decline in seeing young women with genital warts. It’s been significant over the past – I would say even just the past 10 years. – nurse practitioner*	*Characteristics of Individuals*: Knowledge and beliefs about the intervention	*Inner setting*: Culture (Recipient-Centeredness)
*Oh, the benefits to the clinic, yeah. We would have a lot more, I would say, negative results as far as HPV goes. I haven’t I’m trying to think – there aren’t very many patients that I have seen who have tested positive for HPV. So, that may be showing how effective the vaccine actually is. – nurse*	*Characteristics of Individuals*: Knowledge and beliefs about the intervention	*Innovation* Characteriscs
*I think because of how the HPV was first rolled out as for women, that it kinda stayed that way and then I think because we linked it so heavily with cervical cancer, and the boys were all ready behind when we started doing that and then they just got forgotten about. I mean I think it’s better now. I think pediatricians are better than they were at the beginning. […]And definitely boys that – men, I should say, men that were not vaccinated as teenagers, they’re not interested. – nurse practitioner*	*Characteristics of Individuals*: Knowledge and beliefs of the intervention	*Individuals*: Innovation Recipients
*Not much, to be honest. I think they might think cervical cancer. The boys definitely are not thinking at all that it applies to them. They do think it’s preventing an STD more than the cancer. So, we can kind of push the genital warts issue. It’s a little harder for us to push the cancer issue, they don’t see that as much as a threat to them. – nurse practitioner*	*Characteristics of Individuals*: Knowledge and beliefs of the intervention	*Individuals*: Innovation Recipients
*I do think that’s a very important thing to highlight though with the number of people who have HPV-related oropharyngeal cancer each year is very high. And it’s male. – physician (OBGYN)*	*Characteristics of Individuals*: Knowledge and beliefs of the intervention	*Individuals*: Innovation Recipients
*I did my residency training in [southeastern state]. So, in [southeastern state], I took care of a lot more children in the age group of 9 to 18. And the parents there were pretty open to getting the vaccination. What I found here [south state] is I’ve had a lot of patients, who are like 18, and they came down here for college, and they don’t remember if they got an HPV shot. So, I’ll either get their records from before and then I’ll let them know if they’re due, or if they know that they haven’t gad it generally, they want it. – family physician*	*Characteristics of Individuals*: Knowledge and beliefs of the intervention	*Individuals*: Innovation Recipients
*I think more know about the cervical cancer, but I am just finding a lot of kids not knowing about the oral cancer component of it. And kind of more of a focus that it’s highly recommended for men, especially men that have sex with men, as far as the anal cancers too. Because I think in the beginning it was just targeted for women, then it got the approval for men, so just trying to say that it’s equally important for both. – family physician*	*Characteristics of Individuals: Knowledge and beliefs of the intervention*	*Individuals: Innovation Recipients*
*Unfortunately, a lot of it’s on our end as far as just being too busy, not remembering, if there’s an acute problem in progress, so somebody’s sick, then I’m not gonna say, ‘And by the way, would you like the HPV vaccine? ‘ And these students only come to us when they’re kind of in extremis anyway. So, that’s a barrier. – physician*	*Characteristics of Individuals*: Knowledge and beliefs of the intervention	*Individuals*: Innovation Recipients
*Sometimes we even have, even though the majority of our patients are over 18, we still have a lot of parents that are against it. So, I feel like sometimes some of our patients have to kind of go behind their parent’s back to make the decision to get that vaccine. – nurse practitioner*	*Characteristics of Individuals*: Knowledge and beliefs about the intervention	*Process*: Assessing context
*I think also to check to see, just not assume but everyone think that adolescents got that because if their parents were against it and now, they’re adults, it’s a good opportunity to try to retrain the person that was given wrong information by their parents. So, those two things are big for providers they need to be aware of. – nurse practitioner*	*Characteristics of Individuals*: Knowledge and beliefs about the intervention	*Process: Assessing context*
*It’s [HPV vaccine hesitance] because of, I think, what their parent may want or may not want them to have, because their parent may not know that they are sexually active, and if they engage in a conversation, mom’s afraid that I’m going to then become sexually active. But that’s a very, very small amount, but there is still that fear that if they get the vaccine, then they’re going to become sexually active. But it’s, like I said, a very small amount. – nurse practitioner*	*Characteristics of Individuals*: Knowledge and beliefs about the intervention	*Process*: Assessing context
**PROCESS** *(e.g., activities and strategies used to implement vaccination)*		
*R: So, what are some things that you think might get in the way of providers recommending the HPV vaccine to their patients?* *P: Patient overload burnout […]. – nurse practitioner*	*Process*: Executing	*Inner setting*: Work Infrastructure
*Yeah. So, then we have to figure out – and sometimes, it’s getting easier to figure out their previous vaccine history. There’s a lot more interfacing with outside databases that we can then look up, or they bring their records, and we enter them into our database so that we can help track their vaccination that way. - physician*	*Process*: Executing	*Inner setting*: Structural Characteristics (Information technology infrastructure)
*Everyone always complains, “Oh, we don’t have time for this. You guys want us to do this.” So, I think creating a way to make it easier might propel providers to want to participate. – nurse practitioner*	*Process*: Executing	*Inner setting: work infrastructure*

## Data Availability

De-identified quantitative data from this study are available from the corresponding author upon reasonable request. Given the small sample size and the qualitative nature of the aims, data are not being posted in a repository in order to protect the confidentiality of the study participants.

## Highlights

Human papillomavirus (HPV) vaccination is a cancer prevention strategy. The current manuscript provides insights about barriers and facilitators to HPV vaccination among young adults which can inform intervention strategies, including patient and provider education.

## Code Availability

NVivo software used for the qualitative analyses is available via QSR international, and Stata software used for quantitative analyses is available via StataCorp LLC.
